# Supplementary material for: An electrophysiological marker of arousal level in humans
Source: eLife. 2020 Jul 28;9:e55092. doi: 10.7554/eLife.55092 (PMC7394547; doi:10.7554/eLife.55092)
Supplement: Transparent reporting form [file elife-55092-transrepform.docx]

***eLife’s* transparent reporting form**

We encourage authors to provide detailed information *within their submission* to facilitate the interpretation and replication of experiments. Authors can upload supporting documentation to indicate the use of appropriate reporting guidelines for health-related research (see [EQUATOR Network](http://www.equator-network.org/%20)), life science research (see the [BioSharing Information Resource](https://biosharing.org/)), or the [ARRIVE guidelines](http://www.plosbiology.org/article/info:doi/10.1371/journal.pbio.1000412) for reporting work involving animal research. Where applicable, authors should refer to any relevant reporting standards documents in this form.

If you have any questions, please consult our Journal Policies and/or contact us: [editorial@elifesciences.org](mailto:editorial@elifesciences.org).

**Sample-size estimation**

- You should state whether an appropriate sample size was computed when the study was being designed
- You should state the statistical method of sample size computation and any required assumptions
- If no explicit power analysis was used, you should describe how you decided what sample (replicate) size (number) to use

Please outline where this information can be found within the submission (e.g., sections or figure legends), or explain why this information doesn’t apply to your submission:

No explicit power calculations were performed to determine sample sizes. For intracranial patients we aimed for 10 patients per group as this sample in line with previously published work. The EEG data sets had been collected for previous studies and were reanalyzed.

The following numbers of patients/ participants were analyzed for the current study:

Sleep EEG - healthy participants, n=20, mean age 20.4, 12 female;

Sleep intracranial EEG - epilepsy patients, n=10, mean age 33.1 , 6 female; Anesthesia EEG - normal hospital population, n=10, mean age 53.3, 2 female; Anesthesia intracranial EEG - epilepsy patients, n=12, mean age 26.6, 3 female.

**Replicates**

- You should report how often each experiment was performed
- You should include a definition of biological versus technical replication
- The data obtained should be provided and sufficient information should be provided to indicate the number of independent biological and/or technical replicates
- If you encountered any outliers, you should describe how these were handled
- Criteria for exclusion/inclusion of data should be clearly stated
- High-throughput sequence data should be uploaded before submission, with a private link for reviewers provided (these are available from both GEO and ArrayExpress)

Please outline where this information can be found within the submission (e.g., sections or figure legends), or explain why this information doesn’t apply to your submission:

This study used four independent data sets (sleep EEG, sleep intracranial EEG, anesthesia EEG and anesthesia intracranial EEG) and replicated the effect in all four separate cohorts.

Data was excluded from analysis for the following reasons: Noisy, white matter and epileptic channels were excluded after visual inspection (see Data preprocessing). One patient in the anesthesia EEG group was excluded because anesthesia began before recording started.

This information can be found in the figure legends, in the results and Material and Methods part of the paper.

**Statistical reporting**

- Statistical analysis methods should be described and justified
- Raw data should be presented in figures whenever informative to do so (typically when N per group is less than 10)
- For each experiment, you should identify the statistical tests used, exact values of N, definitions of center, methods of multiple test correction, and dispersion and precision measures (e.g., mean, median, SD, SEM, confidence intervals; and, for the major substantive results, a measure of effect size (e.g., Pearson's r, Cohen's d)
- Report exact p-values wherever possible alongside the summary statistics and 95% confidence intervals. These should be reported for all key questions and not only when the p-value is less than 0.05.

Please outline where this information can be found within the submission (e.g., sections or figure legends), or explain why this information doesn’t apply to your submission:

Statistical analysis methods are described in the results, the figure legends and in detail in the Material and Methods part of the paper, section Statistical testing.

Single subject examples can be found in Fig. 1a and 2a and Fig. 3.

(For large datasets, or papers with a very large number of statistical tests, you may upload a single table file with tests, Ns, etc., with reference to sections in the manuscript.)

**Group allocation**

- Indicate how samples were allocated into experimental groups (in the case of clinical studies, please specify allocation to treatment method); if randomization was used, please also state if restricted randomization was applied
- Indicate if masking was used during group allocation, data collection and/or data analysis

Please outline where this information can be found within the submission (e.g., sections or figure legends), or explain why this information doesn’t apply to your submission:

Patients were recruited consecutively for sleep intracranial EEG, anesthesia EEG and anesthesia intracranial EEG.

The sleep EEG participants were recruited consecutively from healthy undergraduates at UC Berkeley. Randomization was not relevant to this study as it was purely observational and did not interfere with regular patient care. Patients were recruited consecutively in each sub-study.

Blinding was not relevant to this study as it was purely observational and contained no intervention. Data was collected at different centers: Anesthesia EEG and intracranial EEG (Oslo University Medical Center, Norway), sleep EEG (UC Berkeley), sleep intracranial EEG (UC Irvine Medical Center).

**Additional data files (“source data”)**

- We encourage you to upload relevant additional data files, such as numerical data that are represented as a graph in a figure, or as a summary table
- Where provided, these should be in the most useful format, and they can be uploaded as “Source data” files linked to a main figure or table
- Include model definition files including the full list of parameters used
- Include code used for data analysis (e.g., R, MatLab)
- Avoid stating that data files are “available upon request”

Please indicate the figures or tables for which source data files have been provided:

Source data files for the main figures are provided on Dryad.
